# Supplementary material for: Evaluation of Parabens and Bisphenol A Concentration Levels in Wild Bat Guano Samples
Source: Int J Environ Res Public Health. 2023 Jan 20;20(3):1928. doi: 10.3390/ijerph20031928 (PMC9916121; doi:10.3390/ijerph20031928)
Supplement: Supplementary file 1 [file ijerph-20-01928-s001.zip › ijerph-2107858-supplementary.pdf]

Supplementary materials for

Evaluation of parabens and bisphenol A concentration levels in wild bat guano samples

Slawomir Gonkowski[1], Julia Martín[2], Irene Aparicio[2], Juan Luis Santos[2], Esteban Alonso[2], Liliana Rytel[3]

[1]Department of Clinical Physiology, Faculty of Veterinary Medicine, University of Warmia and Mazury, Street Oczapowskiego 14, 10-719 Olsztyn, Poland.

[2]Departamento de Química Analítica, Universidad de Sevilla, C/ Virgen de África, 7, E-41011, Sevilla, Spain.

[3]Department of Internal Diseases with Clinic, Faculty of Veterinary Medicine, University of Warmia and Mazury in Olsztyn, ul. Oczapowskiego 14, 10-719, Olsztyn, Poland.

Corresponding author: Liliana Rytel, e-mail: liliana.rytel@uwm.edu.pl

Liquid chromatography–tandem mass spectrometry conditions

Chromatographic analyses were performed on an Agilent 1260 Infinity II (Agilent, Santa Clara, CA, USA). Compounds were analysed using a LC method previously published by Martín et al. (2017) slightly modified. Separation was carried out using a HALO C18 Rapid Resolution (50 x 4.6 mm i.d., 2.7 µm). The mobile phase was composed of methanol (solvent A) and a buffer solution acetic acid/ammonium acetate (pH 4.4) (solvent B). The elution program was as follows: 0-14 min, linear gradient from 28 to 70% of solvent A, increased to 80% of A in 5 min and to 100% of A in 6 min and held for 2 min. Flow rate was 0.6 mL min^{-1}.

The LC system was coupled to a 6495 triple quadrupole mass spectrometer with electrospray ionization (ESI) source operated in negative mode. Two MRM transitions, for identification and quantification purposes, were selected for each analyte. Fragmentation conditions are summarized in Table S1.

Table S1. MRM conditions used for LC-MS/MS of parabens and BPA.

Compound	Acronym	Internal standard	MRM 1	MRM 2	Fragmentor (V)	Collision Energy (eV)	Ionization mode
Methylparaben	MeP	PrP-$^{13}C_6$	151.2 > 92.1	151.2 > 136.1	160	16	ESI-
Ethylparaben	EtP	PrP-$^{13}C_6$	165.2 > 92.1	165.2 > 137.1	160	20	ESI-

Propylparaben	PrP	PrP-$^{13}C_6$	179.2 > 92.1	179.2 > 136.1	160	24	ESI-
Butilparaben	BuP	PrP-$^{13}C_6$	193.1 > 92.1	213> 136.1	160	24	ESI-
Bisphenol A	BPA	BPA-d_{16}	227.3 > 133.0	227.3 > 211.8	160	32	ESI-
Propylparaben-$^{13}C_6$	PrP-$^{13}C_6$	-	185.2>99.1	185.2>98.0	160	24	ESI-
Bisphenol A-d_{16}	BPA-d_{14}	-	241.2>141.9	241.2>223	160	28	ESI-

MRM 1: transition used for quantification; MRM 2: transition used for confirmation.
